# Progress in Surgery

**Published:** 1894-12-29

**Authors:** 


### Progress in Surgery.

### GENERAL SURGERY.

**Syphilitic Pleurisy.**—Twelve cases of this affection have been collected by Chantemesse,[1] including two of his own. In both the latter there were discovered the physical signs of pleurisy with effusion, and in one aspiration yielded a quantity of straw-coloured fluid. Both had *râles,* due probably to an eruption of roseola on the bronchial mucous membrane. M. Chantemesse used the intra-venous method of Bacelli, but would prefer intra-muscular injections of the red oxide of mercury dissolved in sterilised olive oil. The prognosis is, as a rule, favourable.

**Syphilis.**—Price[2] gives some cases of syphilis treated with sulphate of copper. He claims: (1) That copper exercises a specific action in syphilis, which is especially directed towards the lymphatic system. It is for this reason more radically curative than mercury. (2) It is slow in removing skin symptoms of the secondary stage. (3) It prevents the development of mucous patches and throat symptoms. (4) It is a very active drug, and it is wise to omit its use one day in the week, if not more frequently. The signs of injurious action are first a voracious appetite, gradually followed, if dose be not reduced or drug discontinued, by prostration, giddiness, pallor, rapid and weak pulse. (5) The average dose is one-thirtieth of a grain ter die. It is better to give it with the sulphate of iron in pill or solution. (6) This dose is absolutely dangerous in cases of syphilitic cachexia, producing excessive prostration. An excessively small dose must here be given to establish tolerance.

**Syphilis of Pharynx.**—Five cases have been reported by Santi[3] from the Clinic of de Havilland Hall, in which very severe tertiary syphilitic ulcers of the pharynx yielded quickly to swabbing with a solution of copper sulphate, 20 grains to the ounce. The foul surface should be first sprayed with an alkaline solution and wiped as clean as possible with pledgets of wool on holders, next painted with a solution of cocaine (20 grains to the ounce) and lastly thoroughly painted with the copper solution. This treatment is renewed daily, or every other day, the pharynx being kept clean by some simple gargle.

**Syphilis of the Epididymis** is declared by C. W Allen[4] to be more frequent in the secondary stage than has been believed, and this without the body of the testis being affected. It is especially in the beginning of the secondary period that it will be found if sought for. It occasions no discomfort, and has a marked tendency to spontaneous retrogression. He thinks, however, its presence declares a rather pronounced attack of syphilis. The induration is located in the head of the organ, and is extremely hard. The importance of this in relation to tubercular disease will be apprehended. The chronic enlargement so often left after gonorrhœal epididymitis involves mostly the tail of the organ, while syphilis chiefly implicates the head.

**Orbital Periostites.**—Trousseau has recorded[5] a very rare case of orbital periostitis due to syphilis, remarkable on account of the intensity of the cerebral phenomena, which suggested tubercular meningitis.

**Genito-Urinary Surgery.**—Chaput,[6] in a paper on "Inosculation of the Ureter with the Intestine," summarises his views as follows: (1) The anastomosis of the ureter with the intestine is an easy and favourable operation. That it does not lead necessarily to a hydro-nephrosis due to contraction of the orifice of exit, nor to pyelo-nephrosis due to ascending infection, is proved by his personal observations. (2) The bilateral operation, successfully performed by Navaro on dogs, can certainly be successfully performed on man. (3) The exit of urine into the intestine does not cause special inconvenience, and neither impairs digestion nor irritates the mucosa; the stools are somewhat more frequent, but not more so than normal micturition. (4) The operation is valuable where more simple methods are inapplicable.

Willy Mayer,[7] reports a case in which the vesical end of the ureter had been resected with a tumour of the bladder. By the cystoscope a tumour was seen in the bladder of large size. Opening of the right ureter was not seen; the left was visible. Where the right one should have been a small amount of turbid urine escaped at intervals. The conclusion arrived at was that the tumour involved and compressed the right ureter, and had led to pyelitis. On February 16th, 1893, a transverse incision was made, and the bladder opened. The tumour was found to be of the size of an apple, involving the orifice of the right ureter, which was invisible. The tumour was partly shelled out and partly cut out by Paquelin's cautery and the scissors. Patient made a good recovery. Two inches of the ureter at its lower end were removed; in fact, that part which lay obliquely in the wall of the bladder. Cystoscopy seven months later showed a large scar in the bladder at the site of the excision, and the escape of urine out of an irregular oval-shaped hole in centre of it. But urine was still purulent, confirming the diagnosis of pyelitis. There was then no tumour in the right lumbar region.

Ed. Cotterell[8] contributes a paper on the subject of "Calculus of the Ureter." The following are a few of his salient points: After stating that only 13 cases of operative procedure had then been recorded (including two of his own), he points out the futility and danger of palliative treatment with sedatives and antispasmodics, and the overwhelming importance of early operation. The consequences of impacted calculus are four—(1) Complete atrophy of kidney. This only occurs when stone is so impacted that no leakage past it occurs. (2) Hydronephrosis, which may be large. This occurs when the stoppage by the stone is incomplete. Complete atrophy of the kidney is not produced. (3) Pyelitis may be present, and is due to mechanical irritation of pelvis by the stone. This may lead to pyonephrosis or interstitial nephritis. (4) Suppression of urine occurs only from two causes —(*a*) stone impacted in one ureter with atrophy of the other kidney; (*b*) impaction where only one kidney is present. It is of serious importance to physicians not to delay till uræmic symptoms occur, as the early operation is a safe one (only one recorded death), whereas if uræmic poisoning be present, the fatality is great. He points out the absurdity of the theory often given in text-books, that impaction of one ureter may suppress by inhibitive action the secretion of the other kidney. He notes one or two valuable points about symptoms and diagnosis. Renal colic is simulated in that periods of pain intermit with periods of repose. In renal colic the passage of the stone may be mapped out by subjective symptoms, but when impacted, pain radiates from one spot, and on careful palpation between the attacks of pain a tender fixed spot is revealed (a very important point). If this position of tenderness remain constant a valuable guide to diagnosis is furnished.

There are four likely positions of impaction—(1) At junction with the pelvis of the kidney, the stone often enters the upper part of ureter, and the larger part projects into the kidney; (2) where the tube narrows two inches from the top end; (3) at true brim of the pelvis, where the ureter turns forward (4) the junction with the bladder—the favourite place —owing to narrowing of canal.

The shape, size, and smoothness of the stone, of course, also affect the result.

[1] Lancet, July 1, 1894. [2] New York Med. Jour., February 3, 1894. [3] Lancet, June 23, 1894. [4] Med. Record, July 21, 1894. [5] Medical Week, June 22, 1894. [6] Arch. Gen. de Med., January, 1894. [7] Annals of Surgery, July, 1894. [8] Lancet, June 30, 1894.

